# Highly biomimetic spiking neuron using SiGe heterojunction bipolar transistors for energy-efficient neuromorphic systems

**DOI:** 10.1038/s41598-024-58962-3

**Published:** 2024-04-10

**Authors:** Yijoon Kim, Hyangwoo Kim, Kyounghwan Oh, Ju Hong Park, Chang-Ki Baek

**Affiliations:** 1 Department of Convergence IT Engineering, Pohang University of Science and Technology (POSTECH), Pohang, 37673 South Korea; 2 Future IT Innovation Laboratory, Pohang University of Science and Technology (POSTECH), Pohang, 37673 South Korea; 3 Department of Electrical Engineering, Pohang University of Science and Technology (POSTECH), Pohang, 37673 South Korea

**Keywords:** Engineering, Nanoscience and technology

## Abstract

We demonstrate a highly biomimetic spiking neuron capable of fast and energy-efficient neuronal oscillation dynamics. Our simple neuron circuit is constructed using silicon–germanium heterojunction based bipolar transistors (*HBT*s) with nanowire structure. The *HBT* has a hysteresis window with steep switching characteristics and high current margin in the low voltage range, which enables a high spiking frequency (~ 245 kHz) with low energy consumption (≤ 1.37 pJ/spike). Also, gated structure achieves a stable balance in the activity of the neural system by incorporating both excitatory and inhibitory signal. Furthermore, inhibition of multiple strengths can be realized by adjusting the integration time according to the amplitude of the inhibitory signal. In addition, the spiking frequency can be tuned by mutually controlling the hysteresis window in the *HBT*s. These results ensure the sparse activity and homeostasis of neural networks.

## Introduction

Brain-inspired spiking neural networks (SNNs) have emerged as a promising platform for neuromorphic hardware due to their remarkable energy efficiency^1–3^. In SNNs, numerous spiking neurons act as the basic information processing unit of SNNs and transfer signals between synapses. Therefore, spiking neurons with highly compact and energy efficiency are crucial to implement SNNs in hardware. In addition, to enhance the performance of SNNs, several spike-based coding techniques and architectures have implemented biomimetic functions at the neuron level. Inhibition can prevent overfitting of neural networks by suppressing the firing rates of highly activated neurons. This helps the network generalize better to new inputs^4–6^. Another essential function, namely the tunable threshold, can induce sparse activity in SNNs by emulating the brain stimulus activation. This enables dynamic modulation of neural coding precision, potentially saving significant energy by selectively increasing firing rates only at specific times and locations as required^7–9^. In addition, this function provides robust immunity against artificial neurons with threshold deviations, ensuring the homeostasis^10–12^.

The complex neuronal behavior has been emulated through CMOS-based circuits, which typically consist of numerous transistors and capacitors, requiring a large footprint area and power consumptions^13–16^. To overcome these problems, spiking neurons with simple structures have been reported by applying various silicon^17–25^ and non-silicon devices^26–29^. PD-SOI MOSFET based neurons provided a means of incorporating integration and threshold triggering operation using the floating body effect without a capacitor^19–22^. However, these neurons require external circuit for signal conversion and reset process, which results in large power consumption^30^. Recently, the single MOSFET neuron devices have been reported that can realize neuronal behavior without both capacitors and external circuitry. However, these single-device neurons consume large power and have small internal capacitance, limiting their ability to integrate large amounts of synaptic signals^23–25^. Non-silicon devices such as memristors and ferroelectric field effect transistor (FeFET) neurons have also been reported due to their steep switching characteristics and scalable structures. However, these neurons have difficulties in controlling their properties consistently in large-area fabrication. Moreover, the resistance change according to the constant voltage pattern is non-linear, which can make practical application difficult^26–29^. In conclusion, these reported spiking neurons still operate with large energy consumption for periodic neural oscillations incorporating biomimetic functions.

In this work, we proposed a novel spiking neuron using silicon–germanium (SiGe) based heterojunction bipolar transistors (*HBT*s) for low energy applications and implementation of biomimetic functions. Our simple spiking neuron consists of four components (2 *HBT*s, 1 resistor, and 1 capacitor) to realize the periodic integrate-and-fire (IF) behavior without external reset circuit. The latch-up voltage, voltage width and current gain were investigated according to germanium content of *p*-base region. The hetero-bandgap structure of *HBT* amplifies the positive feedback gain of hysteresis in the low voltage range through improved hole storage capability and impact-ionization coefficient. This hysteresis characteristic enables integration, threshold triggering, and self-reset processes to run entirely within a low voltage range, resulting in low spiking energy consumption. The hysteresis properties of *HBT* were utilized to analyze neuronal function for various synaptic inputs. The inhibition of multiple strengths was demonstrated though the control of firing latency achieved by modulation of inhibitory signals. Furthermore, the spiking frequency was tuned by controlling the voltage width of hysteresis of *HBT*s.

## Methods

### Device structure and simulation

**Figure 1:**
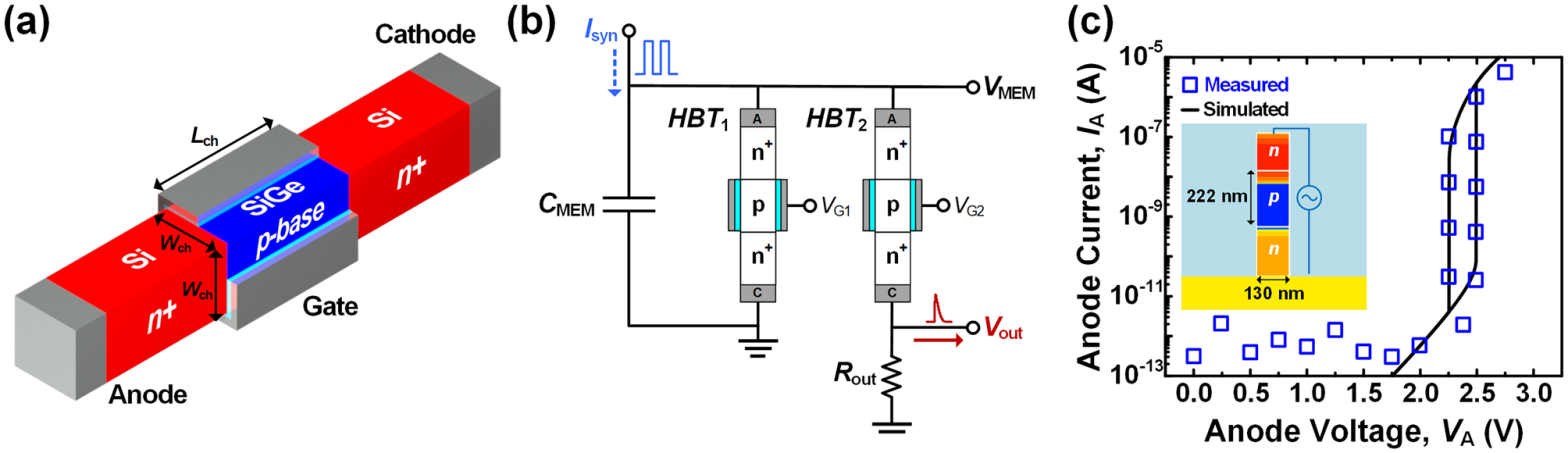
Schematic of (**a**) lateral nanowire-based heterojunction bipolar transistor (*HBT*) and (**b**) diagram of spiking neuron with dual *HBT*s. (**c**) Calibration results fitting on experimental data of the fabricated *biristor*.

Figure 1a shows a schematic of a silicon–germanium (SiGe) based heterojunction bipolar transistor (*HBT*) simulated using the Sentaurus TCAD tool^31^. The *HBT* features a laterally formed silicon nanowire structure with physical *n*^+^-*p*-*n*^+^ layers, where the gated *p*-layer is made of SiGe. The *n*^+^ anode and cathode were heavily doped with 10^20^ cm^–3^. The *p*-base was doped with 5 × 10^17^ cm^–3^, in consideration of impact ionization effect to ensure sufficient supply of holes. The channel length (*L*_ch_) was determined to be 100 nm considering the carrier recombination. A heterogeneous bandgap material, Si_0.6_Ge_0.4_ was utilized in the *p*-base to form hysteresis with high current margin in the low voltage range. The channel area (*W*_ch_^2^) was set to 40 × 40 nm^2^ considering the critical thickness to enable deposition without defects caused by lattice mismatch^32–35^.

Figure 1b illustrates a simple configuration of a spiking neuron designed using TCAD mixed-mode simulations. A membrane capacitor (*C*_MEM_ = 4.7 pF) was used for the integration of synaptic current signals (*I*_syn_). Dual *HBT*s were connected in parallel with the membrane capacitor as a threshold trigger for spike firing and self-reset process. The output resistor (*R*_out_ = 20 kΩ) was employed in series with the *HBT*_2_ to generate the output voltage (*V*_out_).

Figure 1c shows the calibration results of the electrical hysteresis between the simulated and measured data of the *biristor* to ensure the reliability of our simulation. The physics was adjusted using experimental data obtained from fabricated devices^36,37^. Fermi–Dirac distribution and drift–diffusion transport models were used, and the Philips unified mobility model is applied to account for carrier-impurity and carrier-carrier scattering. High-field saturation and doping-dependent mobility models were used, and the Oldslotboom bandgap narrowing model was used to describe the high silicon concentration region. An avalanche generation model was applied to calculate carriers generated by the impact-ionization effect. The doping-dependent Shockley–Read–Hall (SRH) and Auger recombination models were adopted to calculate the recombination rate at the junction surface. A Si-SiGe surface SRH recombination model was also added to consider defects at junctions and interfaces.

### Device characteristics

**Figure 2:**
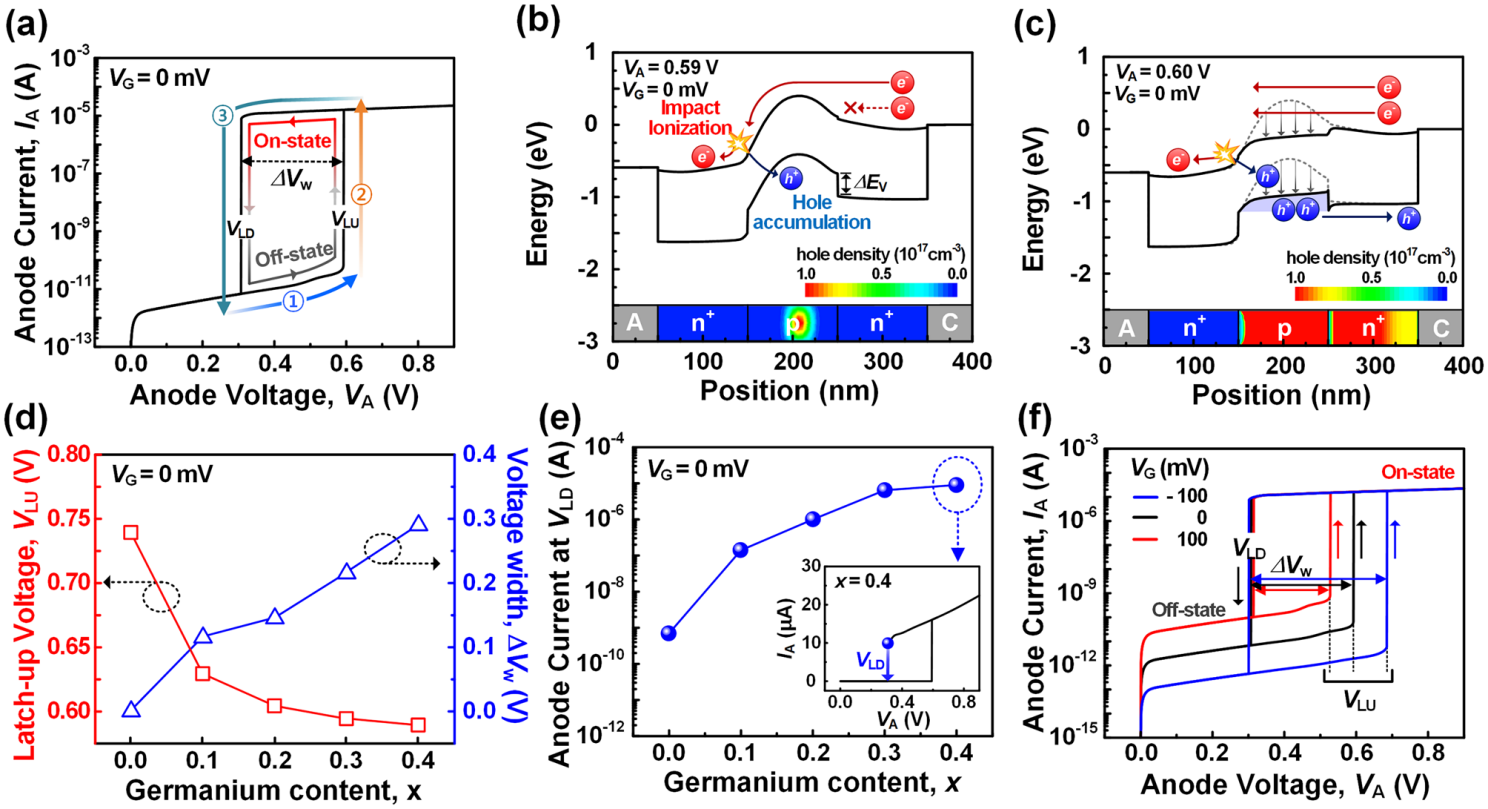
Heterojunction bipolar transistor (*HBT*) (**a**) *I*_A_-*V*_A_ hysteresis at *V*_G_ = 0 mV*.* (**b**) Energy band of *HBT* at *V*_A_ = 0.59 V, where the latch-up phenomenon begins (**c**) Energy band of *HBT* at *V*_A_ = 0.6 V, where the latch-up phenomenon completes (**d**) *V*_LU_ and Δ*V*_w_ as a function of the germanium content (*x*). (**e**) On-state current at *V*_LD_ versus the *x*. (**f**) *I*_A_-*V*_A_ hysteresis at *V*_G_ = –100 mV, 0 mV and 100 mV.

Figure 2a shows the anode current–voltage (*I*_A_-*V*_A_) hysteresis characteristics of the *HBT* under quasi-static conditions when the gate voltage (*V*_G_) is grounded. This hysteresis curve can be seen forming in the low voltage region compared to the typical floating body memory device shown in Fig. 1c. One of the main reasons is that the narrow bandgap of the *p*-base increases the impact ionization coefficient and the valence band offset (Δ*E*_v_) suppresses the diffusion of stored holes. A detailed analysis of this will be illustrated in Fig. 2d following the description of the basic state transition mechanism in Fig. 2b,c. Figure 2b shows energy band of the *HBT* at the latch-up voltage of 0.59 V (*V*_LU_), where the latch-up phenomenon begins. Here, electrons from the cathode are injected into the *p*-base by the applied *V*_A_. These electrons cause impact ionization in the high electric field of the anode-base junction, resulting in the generation of electron–hole pairs. As a result, the potential barrier is lowered by the excess holes stored in the *p*-base. As the potential barrier is lowered, more electrons can be injected into the *p*-base. This series of processes activates positive feedback, which eventually results in abrupt switching of the *HBT* from the off-state to the on-state as shown in Fig. 2c. As *V*_A_ decreases, the on-state is maintained until the latch-down voltage of 0.3 V (*V*_LD_), where the *HBT* rapidly transitions back to the off-state. This is because when *V*_A_ is above *V*_LD_, the impact ionization rate is large enough to maintain a positive feedback loop. Therefore, a counterclockwise hysteresis is formed with a voltage width (Δ*V*_w_ = *V*_LU_-*V*_LD_) of 0.29 V.

It is noteworthy that the hysteresis of the *HBT* exhibits a high current margin of ~ 16 μA at the low *V*_LU_ of 0.59 V. This high current margin in the low voltage region is attributed to the heterogeneous bandgap structure of *HBT*. To investigate the effect of the low bandgap material in the *p*-base, the electrical characteristics in hysteresis curve were extracted according to germanium content (*x* in Si_1-*x*_Ge_*x*_) of the *p*-base (Fig. 2d,e). The analysis range of the *x* was set to within 0.4, which can form a dislocation-free layer, considering the *W*_ch_ of *HBT*. The high current margin in the low voltage region can be explained by amplified *I*_A_ through the increased multiplication factor (*M*) and current gain (*β*) values as follows^37–39^:

1 $$I_{A} = \frac{\beta \cdot M}{{1 - \beta \cdot (M - 1)}}I_{B}$$

The *M* is associated with the impact ionization coefficient that supplies excess holes. Additionally, the *β* is related to the storage capability of excess holes in the *p*-base. As shown on the left axis of Fig. 2d, as the *x* increases from 0.0 to 0.4, the *V*_LU_ decreases from 0.74 to 0.59 V. This is attributed to the increased value of the *M* and *β.* As the *x* increases, the bandgap of the *p*-base narrows and *M* becomes larger. Furthermore, the increased Δ*E*_v_ at the base-cathode junction suppresses the hole diffusion current and increases the value of *β*^40^. Despite the decrease in *V*_LU_, Δ*V*_w_ increases from 0 to 0.29 V, as shown on the right axis of Fig. 2d. This is because our device operates in the low-voltage region, so the effect of *β* is larger than that of *M*, which is greatly amplified at large voltages. Specifically, an increase in the *M* and *β* both reduces the voltage range of hysteresis, but the difference is that the *M* contributes significantly to the reduction of *V*_LU_, while the *β* contributes substantially to the reduction of *V*_LD_. Therefore, the decrease in *V*_LD_ is greater than that of *V*_LU_, resulting in an increase in the Δ*V*_w_.

Figure 2e shows the on-current in *V*_LD_ as a function of the *x*. When the *x* increases, the on-current of the *V*_LD_ is amplified to 10 μA, approximately four orders higher than the value of homogeneous bandgap bipolar transistor. This enhancement is due to the increased positive feedback gain, resulting from the improved value of *M* and *β*. The high current margin of the *HBT* is essential to reliably reset the proposed spiking neuron circuit. Because the self-reset process can be completed as the *HBT* switches rapidly back to the off-state from the on-state, the *HBT* in on-state must discharge the membrane capacitor faster than the charging current signal until the membrane voltage reaches *V*_LD_. Therefore, as can be seen from the simulation results in Fig. 2d, e, the hysteresis with high current margin in the *HBT* is formed in low voltage range. As a result, the *HBT* with this hysteresis ensures the energy-efficient IF operation without the need for external reset circuits.

Figure 2f shows the *I*_A_-*V*_A_ hysteresis characteristics of the *HBT* when the *V*_G_ is − 100 mV, 0 mV and 100 mV. The *V*_LU_ decreases from 0.685 to 0.528 V as the *V*_G_ increases from − 100 mV to 100 mV. A larger *V*_G_ reduces the potential barrier, triggering positive feedback at lower voltage. On the other hands, after latch-up, the stored holes in the *p*-base minimize the effect of *V*_G_ on the potential barrier, so *V*_LD_ maintains nearly constant. This tunable *V*_LU_ modulates the spiking characteristics by adjusting the threshold voltage (*V*_th_) of membrane in IF operation.

### Operation of *HBT* based spiking neuron

**Figure 3:**
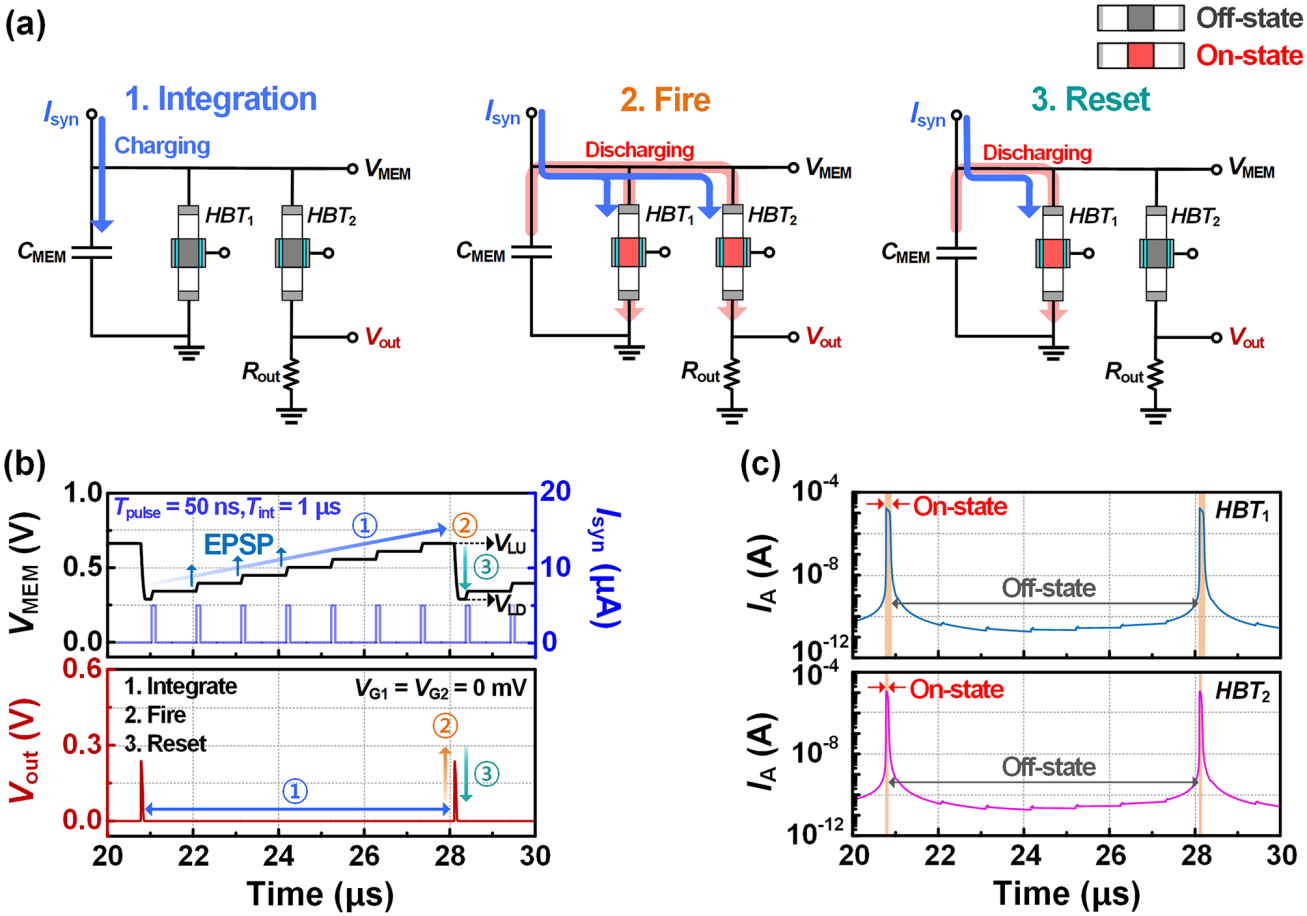
(**a**) Flowchart of integrate-and-fire (IF) operation consisting of three steps: integrate, fire, and reset. (**b**) Periodic IF behavior under excitatory condition (*V*_G1_ = *V*_G2_). (**c**) Anode current of *HBT*_1,2_ corresponding to Fig. 3b.

Figure 3a shows the flow diagram of the integrate-and-fire (IF) operation. One cycle of IF behavior consists of three steps: integrate, fire, and reset. The *I*_syn_ signal is input to the node where *HBT*_1,2_ and the membrane capacitor are connected in parallel. During the integrate-step, the *I*_syn_ signal charges the membrane capacitor, increasing the *V*_MEM_. This increase in *V*_MEM_ corresponds to an excitatory post-synaptic potential (EPSP) in biological terms, which increases the firing probability for post-synaptic neurons. The fire-step occurs after the *V*_MEM_ reaches the *V*_LU_, where *HBT*_1,2_ are converted from the off-state to the on-state. The reduced resistance of *HBT*_2_ leads to an increase in the voltage across the output resistor. This sharp increase in *V*_out_ indicates that the spike is firing. Simultaneously, *I*_syn_ flows through *HBT*_1,2_ without charging the membrane capacitor. The membrane capacitor discharges with the same flow as *I*_syn_, reducing *V*_MEM_. The final reset step is the process where the spike is fully formed and the fire-state transitions back to the initial integrate-state. As the *V*_out_ increases, the potential difference (i.e. *V*_MEM_–*V*_out_) across *HBT*_2_ decreases, which causes the *HBT*_2_ to switch to off-state. This causes *V*_out_ to drop to 0 V and ultimately resulting in the formation of the spike. To achieve periodic IF behavior, both *HBT*_1_ and *HBT*_2_ must be returned to the off-state, which corresponds to the initial integrate-state. *HBT*_1_ discharges the membrane capacitor, reducing *V*_MEM_ to *V*_LD_, which leads to *HBT*_1_ converting itself to off-state.

Figure 3b shows the periodic IF behavior implemented in our spiking neuron from 20 to 30 μs. The *I*_syn_ pulse is 5 μA with the pulse duration (*T*_pulse_) of 50 ns and the interval time (*T*_int_) of 1 μs. When the time was 20 μs, the *V*_MEM_ has reached to 0.68 V due to temporal charging of the membrane capacitor by *I*_syn_ pulses. At the *V*_MEM_ of 0.68 V, the decrease in resistance of *HBT*_2_ causes *V*_out_ to rapidly increase to 0.245 V, namely firing of spike. After *V*_out_ reaches 0.245 V, it begins to decrease corresponding to the decrease of *V*_MEM_. The *V*_MEM_ increases periodically from 0.29 V to 0.68 V when both *HBT*_1,2_ maintain off-state. As shown in Fig. 3c, in the process where *V*_out_ rises and then falls to 0 V (i.e., spike generation), *HBT*_1_ maintains on-state for 50 ns even after *HBT*_2_ transitions back to off-state. Thanks to the large positive feedback gain that can be induced even with small *V*_LU_, *HBT*_1_ ensures stable self-reset in the low voltage range. In addition, *HBT*_1,2_ remain on-state for a short period of time due to their steep-switching characteristics thereby reducing the duration for spike-generation. Accordingly, the hysteresis of the *HBT* allows our spiking neuron to realize high spiking frequency with low-power consumption.

**Figure 4:**
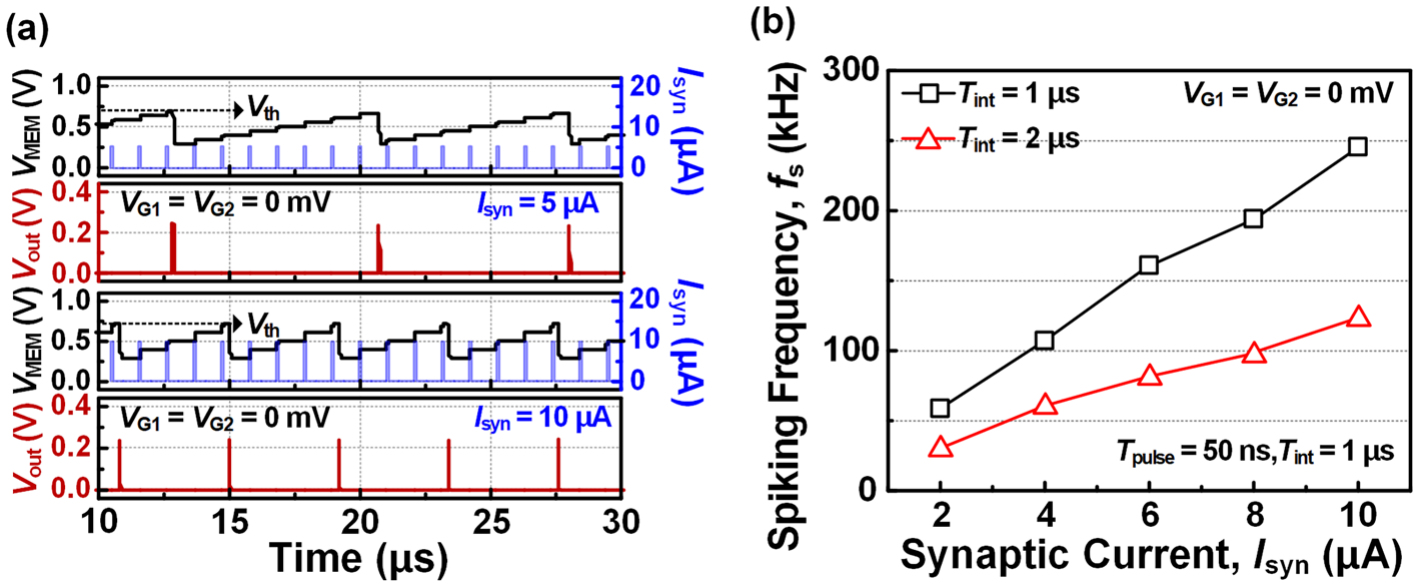
(**a**) Spike response in time domain for synaptic current (*I*_syn_) of 5 μA and 10 μA. (**b**) Spiking frequency (*f*_s_) as a function of amplitude of synaptic current (*I*_syn_) and interval time (*T*_int_).

Figure 4a shows the spike response for different *I*_syn_ values of 5 μA and 10 μA when *T*_pulse_ is 50 ns and *T*_int_ is 1 μs. As *I*_syn_ increases from 5 μA to 10 μA, the spiking frequency (*f*_s_) increases. This is because a larger *I*_syn_ can charge the membrane capacitor faster, which in turn reduces the time required for *V*_MEM_ to reach the *V*_th_. As shown in black line of Fig. 4b, *f*_s_ increases linearly from 58.8 to 245.7 kHz with corresponding to an increment of *I*_syn_ values from 2 to 10 μA. In addition, the impact of *T*_int_ on *f*_s_ is investigated. When *T*_int_ increases from 1 to 2 μs, *f*_s_ decreases by half. The larger the *T*_int_, the less frequently the membrane capacitor is charged by *I*_syn_ pulse, increasing the integration time.

**Figure 5:**
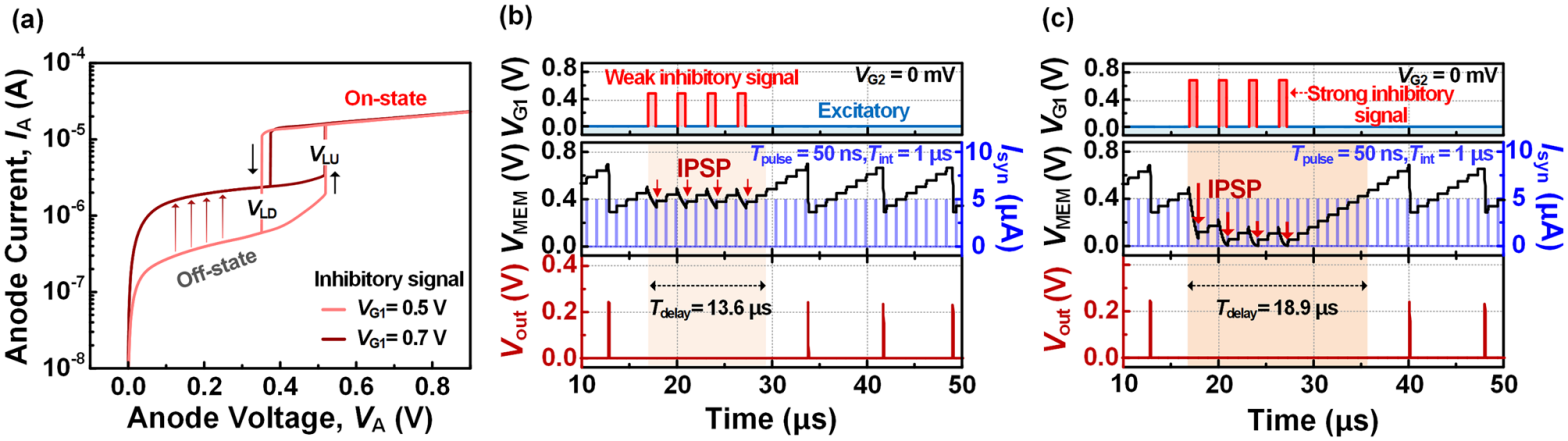
(**a**) *I*_A_-*V*_A_ hysteresis characteristics of *HBT*_1_ under inhibitory signal of *V*_G1_ = 0.5 V and 0.7 V. Neuronal inhibition according to the (**b**) weak inhibitory signals (**c**) strong inhibitory signals.

Multi-strength neuronal inhibitory function can be implemented in the proposed spiking neuron. This function can be induced by applying consecutive inhibitory signals to the gate of *HBT*_1_. Figure 5a shows *I*_A_-*V*_A_ hysteresis characteristics of *HBT*_1_ under inhibitory signal of *V*_G1_ = 0.5 V and 0.7 V. These inhibitory signals increase the off-current of *HBT*_1_, resulting in faster discharge of the membrane capacitor. Consequently, the *V*_MEM_ decreases, corresponding to an inhibitory post-synaptic potential (IPSP) of biological neuron. Figure 5b,c depict the spiking responses resulting from the temporal accumulation of inhibitory and excitatory signals. As shown in Fig. 5b, when an inhibitory signal of 0.5 V is applied, the IPSP suppresses spike firing for 13.6 μs by delaying the *V*_MEM_ from reaching its threshold. Figure 5c shows the intensive neuronal inhibition achieved by the strong inhibitory signal of 0.7 V. This inhibitory signal further accelerates the discharge of the membrane capacitor with a larger off-current of *HBT*_1_, delaying spike firing for 18.9 μs.

**Figure 6:**
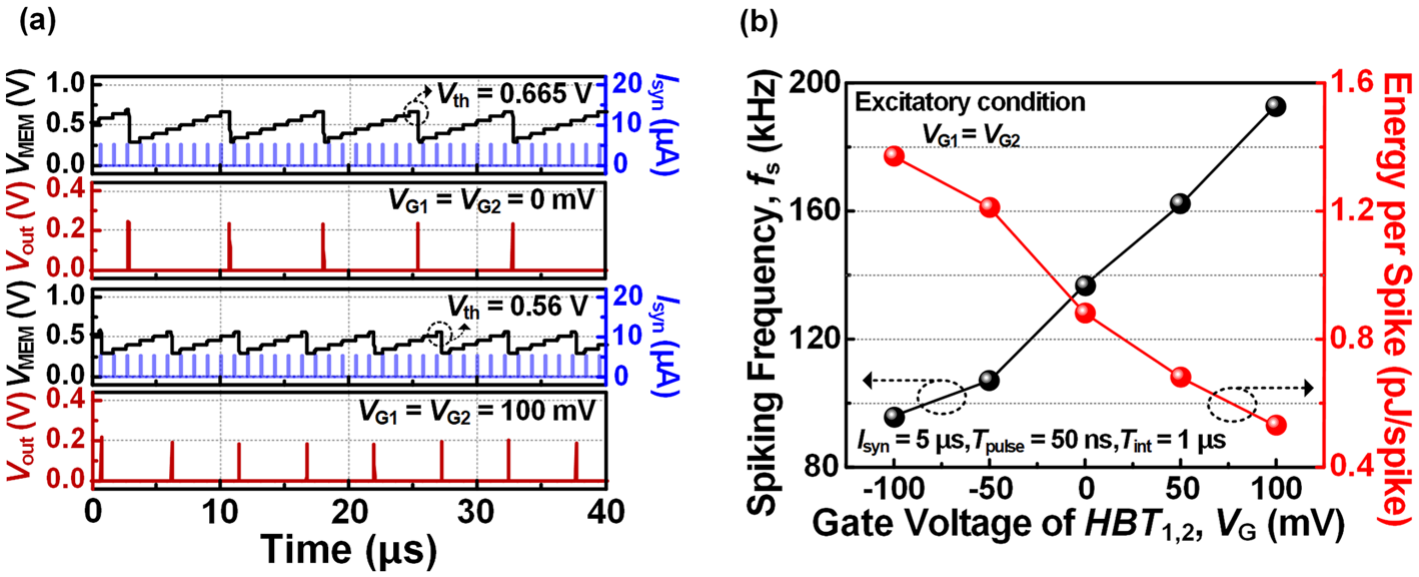
(**a**) Spike response modulated for *V*_G_ values of 0 mV and 100 mV. (**b**) Spiking frequency (*f*_*s*_) and energy consumption per spike as a function of *V*_G_ when an *I*_syn_ pulse of 5 μA is input.

Figure 6a shows the spike response for *V*_G_ values of 0 mV and 100 mV. When the *V*_G_ increases from 0 to 100 mV, the integration time shortens as *V*_th_ decreases from 0.665 to 0.56 V, increasing the number of spikes from 5 to 8 over 40 μs. Additionally, the reduction of *V*_th_ decreases the energy consumed when generating a spike. As shown in Fig. 6b, the *f*_s_ increases from 95 to 192 kHz as *V*_G_ increases from −100 to 100 mV. On the other hand, the energy consumed per spike decreases linearly from 1.37 pJ to 0.53 pJ. Therefore, the spiking properties such as *f*_s_ and energy per spike can be modulated with respect to *V*_G_. These tunable characteristics enable spiking neurons to selectively respond to a specific range of inputs, enhancing the energy efficiency and sensitivity at the neuron level^20^.

Table 1 compares our proposed spiking neuron with previously reported spiking neurons. The comparison focuses on core device, spiking frequency, energy consumption, input type, tunability and circuit components. PD-SOI MOSFET based spiking neurons integrate voltage synaptic signals without capacitor, but requires large energy consumption due to their large threshold (≤ 35 pJ/spike)^19^. JLFET and TBIMOS based neurons also have superior spiking frequency (1 ~ 180 MHz) with ~ 30 times less energy (≤ 1.14 pJ/spike) and ~ 100 times less energy (≤ 0.37 pJ/spike), respectively, compared to PD-SOI MOSFET based neuron^21,22^. PCMO RRAM neurons, another spiking neuron utilizing the capacitor-less integration method, consume low energy (less than 4.8 pJ/spike) to fire spikes^26^. However, in these spiking neurons, which operate the integration step without a capacitor, external reset circuits are essential for periodic IF operation and an *I*-*V* convertor using OP-AMP is also required to receive the current signal from the synaptic device array. In terms of entire spiking neuron circuit, the energy consumption can be significantly increased due to the operation of external circuits that require additional voltage supply^30^. FBFET and FeFET neurons can emulate neuronal behavior without peripheral circuitry but they need a large number of the components (typically 10 and 8) including two capacitors and have high power consumption (≤ 18.8 pJ/spike and ≤ 369 pJ/spike)^18,29^. The integration of both excitatory and inhibitory signals, tunable threshold triggering, and reset operations are fully implemented with a single SOI-MOSFET, however, this neuron device consumes a lot of energy consumption (≤ 45 pJ/spike) and oscillates at low frequency (~ 20 Hz)^23^. Single germanium MOSFET neuron can reduce its threshold voltage, resulting in lower energy consumption (8 pJ/spike) and have a higher spiking frequency (~ 100 Hz) than a single MOSFET neuron^25^. Among these neurons, our proposed simple spiking neuron is particularly capable of achieving periodic neuronal oscillations with a good spiking frequency (~ 245 kHz) and low energy consumption (≤ 1.37 pJ/spike) without external circuit components, while also implementing biomimetic functions such as inhibition and tunable threshold.

**Table 1:** Benchmark comparison of the proposed spiking neuron and various reported spiking neurons.

References	Core device	Spiking frequency	Energy consumption	Input type	Tunability	Circuit components
19	PD-SOI MOSFET	–	35 pJ*	Excitatory	No	1 T + external circuits
21	JLFET	~ 1 MHz	1.14 pJ*	Excitatory	No	1 T + external circuits
22	TBIMOS	~ 180 MHz	0.37 pJ*	Excitatory	No	1 T + external circuits
26	PCMO RRAM	–	4.8 pJ*	Excitatory	No	1 T + external circuits
18	FBFET	–	18.8 pJ	Excitatory	No	8 T + 2 C
29	FeFET	–	369 pJ	Excitatory & Inhibitory	No	6 T + 2 C
23	SOI-MOSFET	~ 20 Hz	45 pJ	Excitatory & Inhibitory	Yes	1 T
25	Ge-MOSFET	~ 100 Hz	8 pJ	Excitatory	Yes	1 T
This work	SiGe-HBT	~ 245 kHz	≤ 1.37 pJ	Excitatory & Inhibitory	Yes	2 T + 1C + 1R

*Exclude the energy consumption of external circuits.

## Conclusions

We have successfully developed a highly biomimetic spiking neuron composed of four components. The heterogeneous bandgap structure of *HBT* results in the formation of hysteresis with high current margin in the low voltage region. By taking advantage of these hysteresis characteristics, the periodic IF behavior can be operated reliably at high frequency (~ 245 kHz) with low energy consumption (≤ 1.37 pJ/spike). Through modulation of inhibitory signals, inhibition is implemented in multiple strengths, thereby effectively regulating excessive firing. Additionally, the threshold can be adjusted to modulate the spiking frequency by controlling the gate bias of *HBT*s. These features play an important role in the sparse activity and homeostasis of neural networks. Consequently, our developed neuron can be a strong candidate for realizing fast and energy-efficient neuromorphic systems.

## Data Availability

The data generated and/or analyzed during the current study are not publicly available for legal/ethical reasons but are available from the corresponding author on reasonable request.
